# Ileosigmoid knotting: A rare cause of acute abdomen with fatal outcome in a 60-year-old man: Case report from a resource-limited setting

**DOI:** 10.1016/j.ijscr.2025.111641

**Published:** 2025-07-09

**Authors:** Dagne Aschenaki Argaw, Wali Ahmed Nur, Musse Ahmed Ibrahim, Mohamed Ayanle Hassan, Aidrose Ahmed Mohamud, Addisu Assfaw Ayen

**Affiliations:** a Department of General Surgery, Garbo Primary Hospital, Somali, Ethiopia; b Bachelor Degree Radiology Technology, Masters on Public Health, Chief Executive Officer, Garbo Primary Hospital, Somali, Ethiopia; c Department of General Surgery, Somali Regional Health Bureau, Jigjiga, Ethiopia; d Master of Public Health, Master of Project Planning and Management and Ph.D Candidate, Somali Regional Health Bureau, Jigjiga, Ethiopia; e Master of Public Health, Master of Project Planning and Management, Somali Regional Health Bureau, Jigjiga, Ethiopia; f Department of Internal Medicine, Debre Tabor University, Debre Tabor, Ethiopia

**Keywords:** Ileosigmoid knotting, Intestinal obstruction, Resource limiting setup, Case report, Ethiopia

## Abstract

**Introduction and importance:**

Ileosigmoid knotting(ISK), a rare but life-threatening condition which first described in 1845. It occurred when the ileum twisting around the sigmoid colon and causing intestinal obstruction and potential perforation.

**Presentation of case:**

A 60-year-old male from the Garbo region of Somali, Ethiopia, presented to the hospital after a a four-day history of intestinal obstruction symptoms. On examination, he was tachycardic and febrile, and exhibited diffuse abdominal tenderness. A diagnosis of intestinal obstruction with generalized peritonitis was made after abdominal x ray. The patient was resuscitated with fluids, started on antibiotics, and a nasogastric tube was placed. Emergency laparotomy revealed ileosigmoid knotting with gangrenous bowel. En bloc resection of the affected ileum and sigmoid colon was performed. An ileo-jejunal anastomosis and an end colostomy were constructed. Despite these interventions, the patient succumbed to uncontrolled sepsis on the fourth postoperative day.

**Clinical discussion:**

Ileosigmoid knotting (ISK), a rare and deadly form of intestinal obstruction, is more common in the “volvulus belt” regions like Africa and parts of Asia. It involves a twisted knot of the ileum and sigmoid colon and can be caused by factors like a narrow mesentery or hypermobile ileum. ISK is difficult to diagnose with standard tests and often requires surgical exploration. The mortality is high (up to 48 %), especially in resource-limited settings due to various challenges.

**Conclusion:**

Ileosigmoid knotting is a rare and dangerous cause of intestinal obstruction requiring quick action. Poor outcomes are common, especially in resource-limited areas like Ethiopia due to late presentation and limited healthcare resources.

## 1 Introduction

Ileosigmoid knotting, a condition first described in 1845 by Parker, is a rare and serious cause of intestinal obstruction and potential perforation [1]. Ileosigmoid knotting occurs when the ileum twists around the sigmoid colon (or its mesentery), or vice versa [2]. Patients with ileosigmoid knotting typically present with an acute abdomen secondary to acute intestinal obstruction [3]. This condition is time-sensitive, as delayed diagnosis and treatment can lead to serious complications such as gangrene, perforation, and ultimately, death. Prompt intervention is crucial [1,3]. Ileosigmoid knotting is more commonly observed in individuals between their 30s and 50s, with a higher prevalence in males compared to females [4]. Its occurrence is also more frequent in regions where sigmoid volvulus is common, such as Africa [4]. Preoperative diagnosis of ileosigmoid knotting is challenging and rarely made [5,6]. This is due to the condition's rarity, nonspecific clinical presentation resembling other causes of acute intestinal obstruction, and the lack of definitive radiological findings [5,6]. As a result, the diagnosis is often established intraoperatively during exploratory surgery [5]. Timely diagnosis and appropriate surgical intervention are critical for improved patient outcomes in ileosigmoid knotting [7]. Conversely, delays in diagnosis and management significantly increase the risk of developing life-threatening complications such as sepsis with septic shock, perforation, peritonitis, and ultimately, mortality [7].

This case report details the presentation of a 60-year-old male from the Garbo region of Somali, Ethiopia, who presented after a four-day history of intestinal obstruction symptoms. He was diagnosed with intestinal obstruction and generalized peritonitis. Exploratory laparotomy revealed ileosigmoid knotting with gangrenous bowel. Despite surgical intervention, the patient died on the fourth postoperative day due to uncontrolled sepsis, highlighting the challenges of managing this condition in resource-limited settings. This case report was prepared in accordance with the revised Surgical Case Report (SCARE) 2025 guidelines [8].

## 2 Case presentation

A 60-year-old male from Garbo, Somali Region, Ethiopia, presents with a 4-day history of: recurrent vomiting, crampy abdominal pain, failure to pass feces or flatus. The day prior to admission, his abdominal pain became persistent, and he experienced worsening abdominal distention, intermittent high-grade fever, and fatigue. No known chronic medical problems, prior similar episodes, or contributory family history reported.

On admission, the patient appeared acutely ill, in pain, and confused, but not in acute cardiorespiratory distress. Vital signs were: blood pressure 90/60 mmHg, heart rate 110 bpm, respiratory rate 26 breaths/min, and axillary temperature 38 °C. Abdominal examination revealed a distended abdomen that was diffusely tender and hyperresonant to percussion. Digital rectal exam revealed an empty rectum and blood on the examining finger; no masses were palpated. The remainder of the physical examination was unremarkable.

**Fig. 1:**
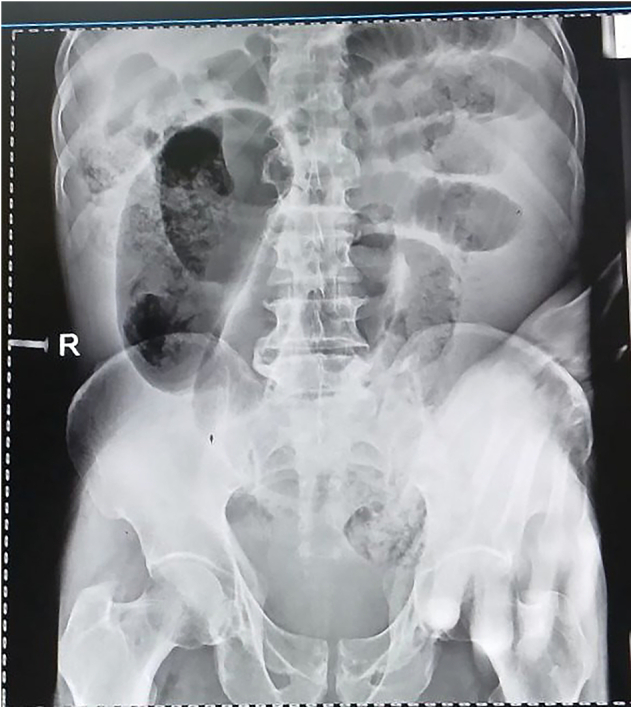
Abdominal X-ray demonstrated marked distention of a large bowel loop extends from the right lower quadrant to the left upper quadrant. Multiple dilated, centrally located small bowel loops with valvulae conniventes spanning their diameter are also noted, consistent with upstream small bowel dilation. No free intraperitoneal air or air-fluid levels are present.

Initial laboratory investigations revealed: WBC 15,000/μL with 85 % granulocytes, hemoglobin 12 g/dL, and platelets 198,000/μL. Blood type was O positive. Organ function tests and serum electrolytes were within normal limits. Abdominal X-ray as (shown in Fig. 1) revealed a hugely distended bowel loop in the right lower quadrant extending to the left upper quadrant. Multiple centrally located dilated small bowel loops with valvulae conniventes spanning the entire luminal diameter were also noted, consistent with dilated upstream small bowel. No free intraperitoneal air was identified. Air-fluid levels were absent.

Based on the clinical presentation and investigations, the patient was diagnosed with acute abdomen secondary to gangrenous small bowel obstruction with generalized peritonitis. Two large-bore intravenous (IV) lines were secured, and resuscitation was initiated with Ringer's lactate. Broad-spectrum intravenous antibiotics were administered, including ceftriaxone 1 g and metronidazole 500 mg. The patient was catheterized, written informed consent was obtained, and he was taken to the operating room for emergency exploratory laparotomy.

**Fig. 2:**
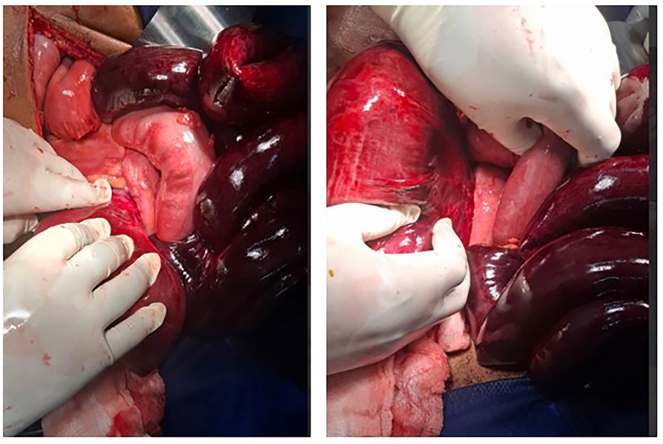
Intraoperative finding revealed an ileosigmoid knot was identified, a complex volvulus in which the distal ileum twisted around the sigmoid colon in a clockwise direction. This resulted in strangulation of both bowel segments, leading to gangrenous changes in the affected ileum and sigmoid colon.

Intraoperative Findings: Approximately 1500 mL of blood‑tinged fluid was present in the peritoneal cavity. An ileosigmoid knot was identified, with the ileum wrapping around the sigmoid colon in a clockwise direction as shown in Fig. 2. Both the ileum and sigmoid colon were gangrenous (as depicted in Fig. 2).

Procedure: En bloc resection of the gangrenous ileum and sigmoid colon with the associated mesentery was performed. An ileo-jejunal anastomosis was created approximately 15 cm proximal to the ileocecal valve, and an end colostomy was constructed.

Following the operation, the patient was transferred to the recovery unit and kept NPO (nil per os). He was placed on maintenance intravenous fluids, and a nasogastric tube (NGT) was maintained for decompression. Ceftriaxone and metronidazole were continued, and adequate analgesia was provided. However, on 3rd post-operative day (POD 3), the patient developed worsening abdominal distention, new-onset fever, and one episode of vomiting. Abdominal ultrasound revealed no intra-abdominal collections. CBC showed worsening leukocytosis (WBC 19,000/μL with 90 % granulocytes) and anemia (hemoglobin 9 g/dL). Platelet count was within normal limits. Serum electrolytes revealed hypokalemia (potassium 3.2 mEq/L); other electrolytes and cardiac troponin were normal. Creatinine increased to 1.5 mg/dL. Antibiotics were revised to cefepime and renal-dose adjusted vancomycin. 40 mEq of potassium chloride (KCl) was added to the maintenance fluids. Despite these measures, the patient's condition deteriorated. He became agitated, fever worsened, and blood pressure dropped. He became hypotensive despite adequate fluid resuscitation, and vasopressor support with epinephrine was initiated and escalated. Despite all interventions, the patient died on postoperative day 4, with a presumed diagnosis of septic shock of gastrointestinal origin. The timeline summary of the patient's course is shown in Table 1 below.

**Table 1:** Timeline of patient's clinical course.

Date	Event
March 01, 2025	Patient developed initial symptoms.
March 05, 2025	Patient visited the hospital.
March 05, 2025	Patient underwent surgery.
March 06, 2025	Patient transferred from recovery to ward.
March 09, 2025	Patient developed worsening of abdominal and systemic symptoms.
March 09, 2025	Antibiotics revised to cefepime and dose adjusted vancomycin; vasopressor imitated.
March 10, 2025	Patient expired.

## 3 Discussion

Ileosigmoid knotting(ISK), also known as compound or double volvulus represents a rare but life-threatening cause of intestinal obstruction associated with a high risk of mortality [5]. It is a type of intestinal knotting where the ileum twists around a sigmoid volvulus (or vice versa), leading to intestinal obstruction and subsequently, bowel ischemia, potentially resulting in patient death [5]; In this case, the patient presented late due to living far from a healthcare facility, leading to the development of significant gangrenous bowel and septic shock, which ultimately resulted in his death.

While ileosigmoid knotting has a global distribution, it is more prevalent in regions known as the “volvulus belt,” including South America, Africa, Eastern and Northern Europe, and parts of Asia and the Middle East [9,10]. These areas have reported a higher incidence of the condition compared to other regions [9]. According to a 2022 update, approximately 1000 cases of ileosigmoid knotting have been reported worldwide [11,12]. This represents about 7.1 % of all sigmoid volvulus cases, with an incidence of 0.3 patients per 100,000 persons per year, or roughly 1.4 patients per year [11]. Ileosigmoid knotting typically affects individuals in their third to fifth decades of life and exhibits a significant male predominance, with reported male-to-female ratios ranging from 2:1 to as high as 6:1 [11].

Limited case reports of ileosigmoid knotting exist in Ethiopia. For example, a 40-year-old female patient diagnosed with ileosigmoid knotting after a 17-h history of symptoms experienced a positive outcome following surgical intervention in 2022 [13]. Another case involved a 38-year-old woman (Gravida VII, Para VI) diagnosed with intestinal obstruction during labor, who also improved after surgery [14]. Furthermore, a case series of 25 ISK patients reported between 2018 and 2023 showed a male-to-female ratio of 2:1 (16:9) [15]. The study found the peak age range for ISK to be between 30 and 50 years, with a mean age of 42.6 years (SD ± 15.9) and a range from 20 to 70 years [15]. A retrospective review of hospital records conducted in Ethiopia from 2018 to 2024 investigated the clinical profile and treatment outcomes of ileosigmoid knotting [16]. The study included 42 patients, although complete data were available for only 38. The mean age of the patients was 39.2 years (SD ± 10.2), with a notable male predominance (M: F = 3.2:1) [16].

Various risk and contributory factors have been identified for ileosigmoid knotting. These include advanced age, the presence of a dolichosigmoid colon (an abnormally long sigmoid colon), a narrow-based mesentery, a hypermobile terminal ileum, and underlying medical conditions such as Parkinson's disease and Alzheimer's disease, as well as poor dietary habits [12]. In the present case, the patient's advanced age was the primary identified risk factor.

The pathogenesis of ileosigmoid knotting often involves the small bowel loops rotating around the narrow base of the sigmoid colon's mesentery. This rotation can be further driven by intestinal peristalsis, creating a closed loop obstruction [17]. While anatomical factors are crucial, other secondary factors like late-stage pregnancy, trans-mesenteric hernias, Meckel's diverticulitis with associated bands, and ileocecal intussusceptions can also contribute to the development of the condition [17].

The clinical presentation of ileosigmoid knotting is often nonspecific, mimicking other causes of acute intestinal obstruction. Common symptoms include abdominal pain, vomiting, abdominal distention, and an inability to pass feces or flatus. These were the initial complaints reported by our patient [1]. However, delayed presentation can lead to serious complications, as seen in our patient who developed fever, altered mental status, significant gangrenous bowel, and peritonitis, ultimately culminating in death despite surgical and antibiotic management [16]. Therefore, early diagnosis, prompt hospital presentation, and timely management are crucial for patients with ileosigmoid knotting to improve outcomes [1].

Diagnosing ileosigmoid knotting can be difficult due to the lack of specific laboratory or imaging features. As with our patient, most individuals are initially diagnosed with general intestinal obstruction. While abdominal CT scans are considered the best imaging modality for diagnosis, they were unavailable in our resource-limited setting [18,19]. The diagnostic accuracy of abdominal CT scans for ileosigmoid knotting exceeds 92 %, demonstrating twisted bowel loops and a whirl sign formed by the mesocolon and twisted loops. Despite this, the method is often limited in resource-constrained settings such as ours, complicating early diagnosis and treatment [20]. Instead, we performed abdominal X-rays, which revealed significantly distended bowel loops and multiple centrally located dilated bowel loops, findings suggestive of intestinal obstruction [18]. These X-ray findings were also similar to those seen in sigmoid volvulus [18]. Given our patient's clinical evidence of peritonitis and the limitations of our setting, barium enema was not an option. While barium enemas can be used in cases of simple intestinal obstruction, serving both as a diagnostic tool for luminal obstruction and a method for decompression in sigmoid volvulus, they are contraindicated if a preoperative diagnosis of ileosigmoid knotting is made [12].

The management of ileosigmoid knotting involves both supportive and definitive treatments. Supportive care includes fluid resuscitation (as in our patient's case, where preoperative hemoglobin was normal who doesnot need blood transfusion), antibiotic administration (our patient received ceftriaxone and metronidazole), and nasogastric tube (NGT) decompression [21]. However, definitive management relies on emergency exploratory laparotomy for both diagnosis and treatment. Surgical options vary depending on the viability of the bowel and the presence of complications like intestinal perforation. If both ileal and sigmoid bowel loops are viable, untwisting the knot after performing an enterotomy may be sufficient [2,12,21]. However, if gangrene is present, as in our patient's case, en bloc resection of both the ileum and colon is necessary [21].

Ileosigmoid knotting carries a high mortality rate, generally reported to be as high as 48 % [22]. However, an Ethiopian six-year review reported a lower mortality rate of 7.9 % [16]. Factors associated with increased mortality include advanced age, pregnancy, comorbidities, and delayed presentation and diagnosis [17]. In the presented case, the patient's advanced age and delayed presentation likely contributed to the fatal outcome. In resource-limited settings like ours, managing ileosigmoid knotting presents several challenges. These include delayed patient presentation due to factors like distance from healthcare facilities, inadequate transportation, and a lack of community awareness. Furthermore, limitations within the healthcare system, such as the absence of ICU care and first-line inotropes, further complicate management. Addressing these challenges requires both national and international collaboration to improve patient outcomes, including raising awareness, enhancing infrastructure, and strengthening hospital care.

## 4 Conclusion

Ileosigmoid knotting is a rare cause of acute intestinal obstruction that can lead to life-threatening complications rapidly. Early patient presentation, diagnosis, and management are essential for positive outcomes. While various factors contribute to poor patient outcomes in general, resource-limited settings face the added challenges of patient-related delays and system-level deficiencies. Therefore, raising community awareness and improving healthcare infrastructure are crucial for improving patient outcomes in these regions. In areas where sigmoid volvulus is prevalent, such as Ethiopia, it is important to maintain a high index of suspicion for ileosigmoid knotting.

## Abbreviations

CBC: Complete Blood Count
ISK: Ileosigmoid Knotting
NGT: Nasogastric Tube
NPO: Nil Per Os
POD: Postoperative day

## Consent

Written informed consent was obtained from the patient's family for publication and any accompanying images. A copy of the written consent is available for review by the Editor-in-Chief of this journal on request.

## Ethical approval

Ethical approval for this study was provided by our institution ethical review committee.

## Guarantor

Addisu Assfaw Ayen.

## Research registration number

N/A.

## Declaration of Generative AI and AI-assisted technologies in the writing process

AI language modelling tools were utilized for the improvement of English-language only in this case report.

## Source of funding

There is no source of funding found for this paper.

## Author contribution

AAA: Conceptualization, design of the study, acquisition of data, drafting the article, revising it critically for important intellectual content, approval of the version to be submitted.

DAA: Analysis, interpretation of data, drafting the article, revising it critically for important intellectual content, approval of the version to be submitted.

WAN: Conceptualization, analysis, drafting the article, revising it critically for important intellectual content, approval of the version to be submitted.

MAI: Acquisition of data, analysis, revising it critically for important intellectual content, approval of the version to be submitted.

MAH: Acquisition of data, analysis, revising it critically for important intellectual content, approval of the version to be submitted.

AAM: Acquisition of data, analysis, revising it critically for important intellectual content, approval of the version to be submitted.

## Declaration of competing interest

All authors declare that they have no conflict of interest.
